# Development of novel molecularly imprinted solid-phase microextraction fibers and their application for the determination of antibiotic drugs in biological samples by SPME-LC/MS^n^

**DOI:** 10.1007/s00216-012-5901-2

**Published:** 2012-03-13

**Authors:** Malgorzata Szultka, Jacek Szeliga, Marek Jackowski, Boguslaw Buszewski

**Affiliations:** 1 Department of Environmental Chemistry and Bioanalytics, Faculty of Chemistry, Nicolaus Copernicus University, Gagarin 7 Street, 87-100 Torun, Poland; 2 Department of General, Gastroenterological and Oncological Surgery, Collegium Medicum in Bydgoszcz, Nicolaus Copernicus University, Joseph Street 53-59, 85-067 Bydgoszcz, Poland

**Keywords:** Solid-phase microextraction, Antibiotic drugs, High-performance liquid chromatography, Mass spectrometry

## Abstract

Novel molecularly imprinted polymer (MIP)-coated fibers for solid-phase microextraction (SPME) fibers were prepared by using linezolid as the template molecule. The characteristics and application of these fibers were investigated. The polypyrrole, polythiophene, and poly(3-methylthiophene) coatings were prepared in the electrochemical polymerization way. The molecularly imprinted SPME coatings display a high selectivity toward linezolid. Molecularly imprinted coatings showed a stable and reproducible response without any influence of interferents commonly existing in biological samples. High-performance liquid chromatography with spectroscopic UV and mass spectrometry (MS) detectors were used for the determination of selected antibiotic drugs (linezolid, daptomycin, amoxicillin). The isolation and preconcentration of selected antibiotic drugs from new types of biological samples (acellular and protein-free simulated body fluid) and human plasma samples were performed. The SPME MIP-coated fibers are suitable for the selective extraction of antibiotic drugs in biological samples.

## Introduction

Determination of chemical compounds from various matrices, including environmental and biological (blood, plasma, urine), is a serious problem in modern analytical chemistry. Chemical compounds analyzed at the ppm and ppb levels require the application of adequate analytical procedures. The most important stage in trace analyses is undoubtedly sample preparation methods. Such sampling methods as the liquid–liquid extraction or solid-phase extraction (SPE) have been known for many years. Samples collection methods, due to their importance, are frequently called *a bottleneck* of the entire analytical procedure (approximately 70% errors). The introduction of solid-phase microextraction (SPME) was the significant step toward the miniaturization development at the end of last century. SPME was reported for the first time by Pawliszyn and coworkers [1] in the early 1990s and is a simple, time-efficient, and solvent-free technique for sample pretreatment. It has been successfully applied to the extraction of various compounds such as biologically active compounds in environmental, food, biological, pharmaceutical, and clinical samples at trace level. SPME is based on the partitioning of analytes between the samples matrix and the polymer film coating [2, 3].

Considering the characteristics of specific selectivity, chemical stability, and easy preparation, molecularly imprinted polymers (MIPs) were proven to be a suitable alternative for selective SPME coating. MIPs are synthetic polymers having a predetermined selectivity for a given analyte or a group of structurally related compounds. The most widely used technique for preparing MIPs is non-covalent imprinting, in which the complex of template and functional monomer is formed in situ by non-covalent interactions. Molecular imprinting becomes increasingly attractive in many fields of chemistry or biology, mainly as a selective sorbent for SPE [4–13]. Recently, the applications of molecularly imprinted materials are also visible and common among the coating for SPME [14–17]. At the beginning, they have been fabricated by modifying MIP-coated silica or monoliths [18, 19]. However, there were some inherent problems such as poor stability, lacking porosity, fragility, low loading capacity, or matrix interferences. On the other hand, molecular imprinting techniques are becoming more commonly accepted as useful methods for the recognition and isolation of key biological target molecules. This attention can be explained by the potential advantages of using molecularly imprinted polymers obtained by electropolymerization, such as their high chemical and thermal stability, low cost, and easy preparation. Additionally, the electropolymerization methods provide a simple and rapid technique of controlling the thickness of the conductive polymer grown adherent to a support of any size and shape. Among them, polypyrrole (PPy) has been one of the most studied materials because it can be used in a neutral pH value, and its stable coating can be easily deposited chemically and electrochemically on various substrate materials [20–22].

Oxazolidinones, the important drugs with linezolid as representative, are widely applied in clinic therapy of bacterial infections, including those caused by resistant organisms. Additionally, they are a novel class of synthetic antimicrobial agents chemically unrelated to any commercially available antibiotics [23, 24]. Linezolid, as the first drug issued from this class, actively responds to Gram-positive bacteria and displays non-bactericidal, time-dependent activity in vitro on staphylococci depending on the binding to the 50S subunit of the prokaryotic ribosome and prevents the formation of the initiation complex for protein synthesis [25, 26]. Daptomycin and amoxicillin, similar to linezolid, are the chemotherapeutics that belong to the different class of antibiotic drugs. Their therapeutic importance is growing with reference to the critical bacterial infections being potentially caused by methicillin-resistant *Staphylococcus aureus*. Additionally, the structure of amoxicillin is quite similar to linezolid. On the other hand, daptomycin presented in biological samples may interfere with linezolid.

The determination of antibiotic drugs used for bacterial treatment infections in complex matrices such as aqueous solutions or physiological fluids (plasma, whole blood) requires appropriate isolation and preconcentration methods. In qualitative analyses and laboratory practice in life chemistry (bioanalytics, clinical analysis, or medicine), the right selection of susceptible and reproducible methods supporting the final determination and validation is essential [27, 28].

In this contribution, we describe the determination of linezolid as a model compound in an acellular and protein-free simulated body fluid with ion concentrations almost equal to those present in the human plasma. The purpose of this paper was to prepare novel MIP-coated SPME fibers by the improved electrochemical polymerization method with linezolid as template. It should be mentioned that there are no available reports for studying the recognition mechanism of linezolid as a template for extraction by MIP-coated SPME fibers from biological samples. The MISPE method was followed by high-performance liquid chromatography (HPLC) with UV detector and MS detection as well. In comparison with the non-imprinted polymer (NIP)-coated fibers, the MIP-coated fiber extraction efficiency was investigated. The proposed method was applied to the simultaneous monitoring of linezolid in the spiked human plasma samples and synthetic body fluids as well.

## Experimental

### Chemicals

All chemicals and reagents were HPLC or analytical grade. Monomers, pyrrole (98%), thiophene (99%), and 3-methylthiophene (98%) were purchased from Sigma-Aldrich (Schnelldorf, Germany) and freshly distilled before the use. Linezolid (LIN), daptomycin (DAPTO), and amoxicillin (AMOX) were supplied by Pharmacia GmbH (Karlsruhe, Germany), Novartis Pharma GmbH (Nuremberg, Germany), and Bayer Healthcare (Leverkusen, Germany), respectively. Other chemicals of analytical quality and purchased were provided by Merck KGaA (Darmstadt, Germany). Water was supplied with Milli-Q RG apparatus by Millipore (Millipore Intertech, Bedford, MA, USA) in our laboratory. Drug-free human plasma was kindly provided by Nicolaus Copernicus University, Collegium Medicum, Torun (Poland), with Bioethical Commission permission (no. 639/2010).

### Instrumentation

The HPLC 1100 (Agilent, Waldbronn, Germany) was used as the chromatographic system. It consisted of quaternary pump, degasser, an automatic sample injection, and variable wavelength UV–vis detector. Additionally, the chromatographic system was coupled with a mass spectrometer (MS^n^) equipped with an electrospray ionization (ESI) interface and operated Mass Hunter software. Positive-ion selected ion monitoring (SIM) mode was used to detect and verify the chemical and molecular structure of linezolid, daptomycin, and amoxicillin among other chemical individuals in human plasma samples, *m/z* = 338 (LIN), *m/z* = 1621 (DAPTO), and *m/z* = 366 (AMOX) that correspond to [M + H]^+^.

For sample evaporation, a Labconco CentriVap DNA concentrator (Kansas City, KS, USA) was used.

In the electroplymerization process, a home-made set-up system coupled with a high-performance potentiostat/galvanostat PGSTAT128N series Autolab model (Utrecht, The Netherlands) was applied.

The chemical and mechanical experiment stability were set up with Optical Stereomicroscope model SZX16 (Olympus, Tokyo, Japan) equipped with a CCD camera and CELL software.

A micrograph of the SPME coatings was obtained from scanning electron microscope (SEM) type 1430 VP (LEO Electron Microscopy, England).

Fourier transform infrared spectroscopy (FT-IR) was recorded with the use of Spectrometer Spectrum 2000 (Perkin Elmer, Waltham, USA) and polymers in KBr pellets.

Water was purified with a Milli-Q Purification System (Millipore, Bedford, MA, USA).

### MIP-coated SPME fiber preparation

As an adsorbent for solid-phase microextraction, the SPME coatings prepared by electrochemical polymerization were used. The procedure of MIP-coated SPME fiber preparation was run based on home-made set-up system connected with potentiostat–galvanostat described earlier [29, 30]. In polymerization, 0.25 M pyrrole (+7 μg/ml LIN), 0.25 M thiophene (+3.5 μg/ml LIN), or 0.1 M 3-methyltiophene (+0.5 μg/ml LIN) solutions in 0.1 M lithium perchlorate (LiClO_4_) in acetonitrile were applied. To perform the polymerization process, dynamic voltamperometry (Linear Sweep Voltammetry-LSV) with threshold potentials from −0.2 to +2.5 V for pyrrole, from −0.2 to +2.7 V for thiophene, and from −0.2 to +3.0 V were used, respectively. Before each polymerization, electrochemical activation of reaction solutions was applied. Stainless steel (SS) applied as a working electrode was washed with water and acetonitrile to remove the impurity and then dried at room temperature before each experiment.

Before each experiment, the bare SS electrode was cyclic-potential-scanned within the potential range −0.2∼1 V in 0.1 M lithium perchlorate solution until a pair of rather well-defined redox peaks was obtained. Then, SS was immersed in the polymerization solution. After deoxygenating the solution by bubbling nitrogen gas for about 20 min, the electropolymerization was performed by cyclic voltammetry in the relevant potential range, with a scan rate on 50 mV/s and seven consecutive scans. After the electropolymerization process, the imprinted polymers were conditioned in 0.1 M NaOH, 0.05 M NaOH (in MeOH), or 0.2 M Na_2_HPO_4_ (in EtOH) using potential cycling between 0 and +1.2 V according to polypyrrole, polythiophene, or poly(3-methylthiophene) MIP-coated SPME fibers, respectively. This process led to removal of the template inside the relevant polymer. Then, the imprinted metallic electrodes were alternately rinsed in an ultrasonic cleaner with alcohol and water, and afterwards dried. A control electrode (non-imprinted polymer modified electrode) was prepared in every case under the same experimental conditions but without adding the linezolid in order to check the reliability of the measurements.

**Fig. 1:**
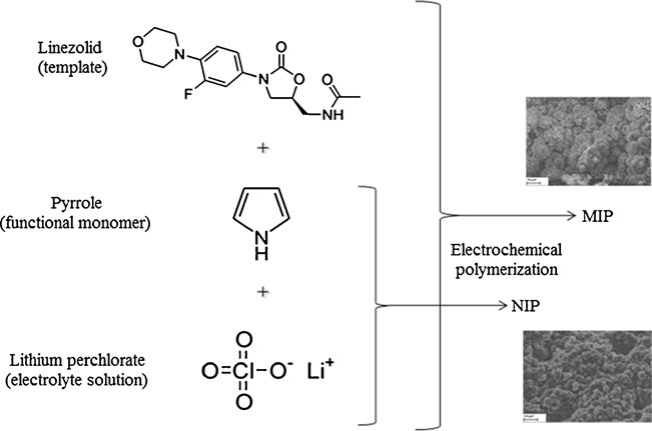
*Scheme* illustrating the MIP-coated SPME fiber synthesis

All the physical–chemical parameters of the applied MIP-coated SPME fibers are listed in Table 1. In addition, the polymerization process is schematically shown in the Fig. 1.

**Table 1:**
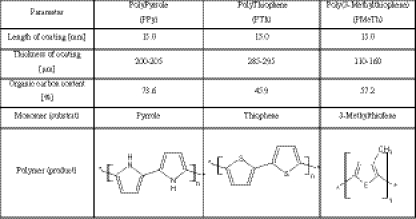
Physico-chemical properties of applied SPME fibers

### Characterization of the MIP-coated SPME fibers

The morphological evaluation of the linezolid MIP-coated SPME fibers was performed by SEM. The infrared absorption spectra (*ν*) of coating between 400 and 4,000 cm^−1^ were obtained in FTIR spectrometer (sample, KBr – 1:100 in mass).

### Acellular and protein-free simulated body fluid samples

Acellular and protein-free simulated body fluid (SBF) used as a biological sample was prepared according to the chemical composition of a human body fluid, with ion concentrations almost equal to these of the inorganic constituents of human body plasma. SBF is known as a metastable buffer solution, and even a small, undesired variance in both preparation steps and storage temperatures may drastically affect the phase purity and high-temperature stability [31]. We discovered that the samples could be stored for a month at 5 °C without any degradation.

### Preparation of stock and standard solutions

The working standard drug solutions, based on the therapeutic concentrations, were prepared by diluting the stock solution of linezolid (125 μg/ml) to a proper volume. The stock solutions were diluted to make working standard solutions in the range from 1 to 20 μg/ml.

The plasma samples were stored at −20 °C. Before the use, plasma was thawed at room temperature and centrifuged at 2,500 rpm for 5 min. The spiked human plasma and SBF solutions were suitable prepared to reach the final concentrations of 1–20 μg/ml.

### Simultaneous SPME of antibiotic drugs from SBF and human plasma samples

MIP-coated SPME fibers were preconditioned in MeOH/water (90:10, *v/v*) for 15 min. The adsorption process of selected antibiotic drug was performed manually (without agitation) in 1.5 ml SBF or human plasma sample during 10 min. Then, SPME fibers were gently washed with distilled water and introduced into 1.5 ml of MeOH/water (50:50, *v/v*) for 5 min. Then, samples after desorption were subjected to HPLC without pre-treatment. The amount of extracted relevant antibiotic drug by SPME coatings was calculated using a calibration curve to acquire the relevant concentration. Total amount of the extracted drug (Am_total_) corresponded to the given equation: ${\text{A}}{{\text{m}}_{\text{total}}} = {{\text{c}}_{\text{des}}} \times {{\text{V}}_{\text{des}}}$, where c_des_ (micrograms per milliliter) is the concentration of drug in the desorption solution, while V_des_ [milliliters] is its volume.

### Chromatographic conditions

#### Linezolid

The chromatographic separation was performed on ACE C8 (150 × 4.6 mm ID, 5 μm particle, 300 Å) column with methanol and water (50:50, *v/v*) mobile phase at a flow rate of 0.45 ml/min. The column temperature was 21 °C, and the injection volume was 15 μl. The detection wavelength was set at *λ* = 251 nm.

#### Daptomycin

The chromatographic separation was performed on ACE C8 (150 × 4.6 mm ID, 5 μm particle, 300 Å) column with acetonitrile (+5 mM ammonium acetate) and water (+5 mM ammonium acetate) (37:63, *v/v*) mobile phase at a flow rate of 0.6 ml/min. The column temperature was 21 °, and the injection volume was 15 μl. The detection wavelength was set at *λ* = 219 nm.

#### Amoxicillin

The chromatographic separation was performed on ACE C18 (150 × 4.6 mm ID, 5 μm particle, 300 Å) column with methanol and water (10:90, *v/v*) mobile phase at a flow rate of 0.6 ml/min. The column temperature was 21 °C, and the injection volume was 15 μl. The detection wavelength was set at *λ* = 230 nm.

### Mass spectrometric detection

The chromatographic system was coupled with a mass spectrometer (MS^n^) equipped with an ESI interface and operated the Mass Hunter software. SIM mode was used to detect and verify the chemical and molecular structure of LIN among the other chemicals in human plasma samples.

Mass spectrometric parameters were optimized for target compound in positive ion mode. An overview of the tandem mass spectrometry (MS/MS) setting is listed in Table 2. Acquisition at full scan and SIM was performed at *m/z* = 338.5 corresponding to [M + H]^+^.

**Table 2:** LC-MS/MS parameters for LIN in human plasma

Parameter	Unit	Setting
Ionspray voltage	[V]	4,000
Capillary temperature	[°C]	320
Scan time	[s]	0.2
Nebulizer pressure	[psi]	30
Gas (nitrogen) flow	[l/min]	8.5
Collision energy	[V]	15
Typical R.T.	[min]	6.9

### HPLC method validation

The proposed method was validated under optimized conditions. Each calibration curve was compiled of points covering from 1 to 20 μg/ml. The accuracy was presented as the ratio of the determined and nominal values of concentrations of the relevant drug and multiplied by 100%. Additionally, the precision was defined as the percentage of standard deviation of the relevant values divided by the average of mean values. The limit of detection (${\text{LOD}} = {3} \times {\text{S}}{{\text{D}}_{\text{xy}}}/b$, where SD_xy_ is the standard deviation and *b* is the slope) and the limit of quantification $\left( {{\text{LOQ}} = {1}0 \times {\text{S}}{{\text{D}}_{\text{xy}}}/b} \right)$ were calculated with the acceptable precision and accuracy according to Konieczka [32]. The recoveries were determined by comparing the peak areas after HPLC measurements of the spiked samples with the direct injection of standard solutions of equal concentrations. Logically, the linearity was obtained by analyzing blank plasma samples or SBF solutions containing standard solutions of drugs at concentrations of 1–20 μg/ml. The concentration range was obtained due to the regression curve $\left( {y = ax + b} \right)$ and correlation coefficient (*R* ^2^). The analytical procedure was fully validated according to the Food and Drug Administration (USA) [33] appropriate guidelines.

## Results and discussion

### Preparation and characteristics of linezolid MIP coating

#### Electropolymerization of molecularly imprinted SPME coatings

**Fig. 2:**
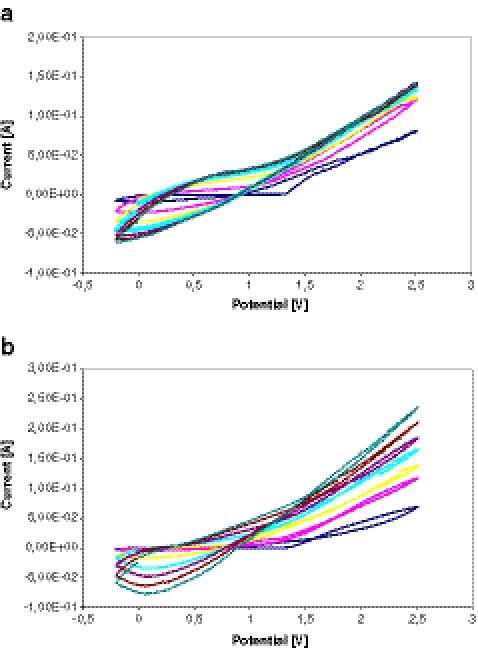
Cyclic voltammograms taken during the electropolymerization of pyrrole (0.25 M): **a** multisweep cyclic voltammograms without and **b** with linezolid (7 μg/ml) onto a SS electrode

The electrochemical behavior of pyrrole, thiophene, and (3-methylthiophene) was investigated in aqueous solution of 0.1 M LiClO_4_ using relevant potential cycling described in “MIP-coated SPME fibers preparation” (versus Ag/AgCl). Figure 2 shows typical cyclic voltammograms for the electropolymerization process of pyrrole on the SS electrode surface (working electrode) in the present of linezolid (template).

Figure 2a demonstrates seven cycles obtained in the same solution. For imprinted electropolymerization, linezolid was added to the electrochemical cell at a concentrations of 7, 3.5, and 0.5 μg/ml for PPy, polythiophene (PTh), and poly(3-methylthiophene) (PMeTh) MIP-coated SPME fibers. Figure 2b demonstrates cyclic scans of electropolymerization of pyrrole in the presence of linezolid.

A cathodic peak was observed at −0.85 V on the first negative scan. Then, the peak current decreased dramatically under continuous cyclic scan. The reductive peak appeared completely irreversible. Finally, the peak current almost approached to zero after seven-cycle scans. The results indicate the formation of PPy coating on the surface of SS electrode, which hinder the monomer further access to the SS electrode surface.

#### Effect of the template concentrations

**Fig. 3:**
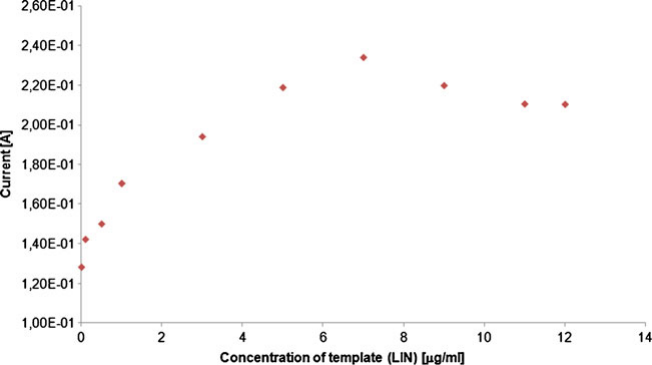
Effect of the template concentration to PPy/MIP-coated SPME response

The structures of linezolid and other interferents were presented earlier [27, 28]. The effect of the template concentration during the coating electrodeposition is shown in Fig. 3.

At the beginning, as the template concentrations grow in the polymerization solution, the response obtained to linezolid of the MIP electrode is also higher. The response of the MIP electrode to linezolid was found to increase with increasing linezolid concentration between 0.1 and 7 μg/ml (Fig. 3). Then, the small decrease of about 8% in the response of MIP electrode above this concentration occurs. Electrooxidation of the pyrrole monomer occurs at the anode, and the resulting polymer deposits onto the surface of the SS. The formation and growth of the polymer coating can be easily seen in Fig. 2 as well. The peaks due to the oxidation and reduction of the film increase in intensity as the coating grows. During the electropolymerization process, linezolid (template) diffuse towards the surface of the SS and trapped in the polymer matrix as a result of the ability of these molecules to interact with the pyrrole units. The creation of the molecular imprint was favored by the chemical forces of the electroactive template, generating a far higher number of recognition sites than a non-electroactive template. Moreover, if higher concentrations were given for electropolymerization process, an increased number of the imprinted cavities may have been destroyed, giving rise to the poorer selectivity. This imprinting process creates a microenvironment for the recognition of linezolid molecule based on shape selection and positioning of the functional groups. The similar situation was found in case of polythiophene and poly(3-methylthiophene) MIP-coated SPME fibers. Finally, the template concentrations for PPy/MIP (7 μg/ml), PTh/MIP (3.5 μg/ml), and PMeTh (0.5 μg/ml) were applied, respectively.

#### Effect of interferents

The selectivity of the MIP electrode in this work was evaluated in the presence of different interfering molecules like daptomycin and amoxicillin. These substances are present in biological fluids and may interfere with the determination of linezolid through conventional methods. It was found that daptomycin and amoxicillin cause negligible changes in the linezolid response even if analyte interferent concentration is on the same level or higher during sample preparation (“Human plasma samples”). This means, the electropolymerized–molecularly imprinted SPME coating can recognize the linezolid molecules by means of shape selection and the size of functional groups. However, the response of linezolid at the NIP electrode is affected by the interferents such as proteins, as what was earlier described in Szultka et al. [27].

#### Reproducibility of the MIP electrode

The reproducibility of the molecularly imprinted SPME coatings was investigated for 15 μg/ml linezolid solution when the HPLC measurements were used. The linezolid peak area response was determined with six SPME fibers; all were produced under the same conditions. The response peak intensity showed a relative standard deviation of approximately 5%, confirming that the results are reproducible.

The chemical stability and robustness of the SPME coatings were confirmed after treatment in different solvents (water, methanol, tetrahydrofuran, chloroform, acetonitrile, MeOH/H_2_O; 50/50 (*v/v*), 2-propanol, MeOH/CH_3_COOH (pH = 4.9), and MeOH/NH_3_ (pH = 8.6)), and there was no significant difference in the porous surface of applied SPME fibers. The MIP- or NIP-coated SPME fibers were immersed in relevant solution for 24 h. The results revealed that MIP or NIP coating remained a good surface quality. Solvent-resistant investigations have been done by using optical stereomicroscope.

#### Characterization of the fibers

##### SEM measurements

**Fig. 4:**
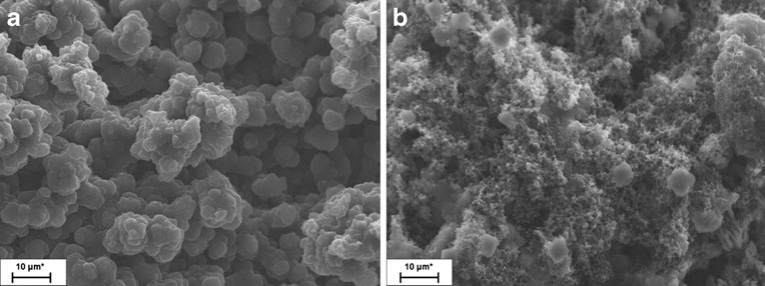
Exemplary SEM micrographs of LIN imprinted polymer (PPy/MIP) (**a**) and non-imprinted polymer (**b**)

SEM was used for the characterization of synthesized NIP- and MIP-coated SPME fibers. Structural variations in the surface morphology and internal structure of both MIP and NIP polymer particles resulting were studied. As shown in Fig. 4, the polymeric material prepared without the LIN demonstrates a much finer structure than the one prepared with template. The results indicated that the existence of LIN would have influenced not only the interaction between the templates and monomer but also the morphology of the polymers. LIN binding with pyrrole during MIP synthesis produced bigger globules and pores in comparison with NIP molecular imprinting without template. The non-imprinted polymer had very small surface globules. NIP- and MIP-coated SPME fibers for polypyrrole have a thickness in range 200–205 and 305–315 μm, respectively.

The similar situation occurred in case of polythiophene or poly(3-methylthiophene) MIP- and NIP-coated SPME fibers (Fig. 4).

##### FT-IR spectroscopy

**Fig. 5:**
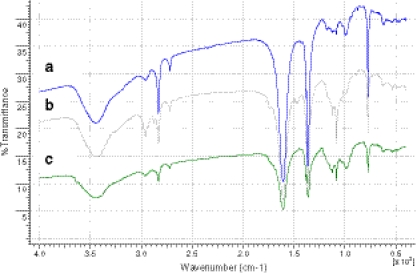
Infrared spectrogram of LIN imprinted polymer (PTh/MIP) before (**a**) and after (**b**) electropolymerization; non-imprinted polymer (PTh) (**c**)

The molecular self-assembly of template-monomers is a premise for the fabrication of imprints. A rapid and valuable tool to evaluate the interaction between the functional monomer and the template in solution was the spectroscopic method. The infrared spectra were performed for the polymer samples in the form of KBr pellets (1 wt.% of polymer). The interpretation spectra were made based on infrared (IR) spectra tables and general information available in the literature as well [34]. The FTIR spectra of polymer in KBr pellet is shown in Fig. 5. The spectra demonstrate bands of the ClO_4_^-^ in doped PTh. The intensive broad band appearing at the wavenumber $\overline {\text{u}} = {3,45}0\;{\text{c}}{{\text{m}}^{{ - {1}}}}$ corresponds to stretching vibrations of –O–H groups. The multiplet between $\overline {{v}} = {2,964}\,{\text{and}}\,{2,876}\;{\text{c}}{{\text{m}}^{{ - {1}}}}$ corresponds to stretching vibrations of the hydrogen attached to the thiophene ring. The intensive signal at $\overline {{v}} = {1,648}\;{\text{c}}{{\text{m}}^{{ - {1}}}}$ is associated with C=C bonds starching vibrations in the PTh ring. Another relatively intensive signal present in $\overline {{v}} = {1,384} - {1,327}\;{\text{c}}{{\text{m}}^{{ - {1}}}}$ corresponds to stretching vibrations of the ClO_4_^-^ anion, used in the process of doping the polymer to generate the appropriate load. Another signal confirming the presence of this ion is a multiplet in the range between $\overline {{v}} = {1,122}\,{\text{and}}\,{1,}0{32}\;{\text{c}}{{\text{m}}^{{ - {1}}}}$. Strong signal at $\overline {{v}} = {785}\;{\text{c}}{{\text{m}}^{{ - {1}}}}$ corresponds to deforming in plane vibrations in the PTh ring. Additionally, the multiplet in the range between $\overline {{v}} = {674}\,{\text{and}}\,{623}\;{\text{c}}{{\text{m}}^{{ - {1}}}}$, with much lower intensity, is associated with C–H out-of-plane stretching vibrations.

**Fig. 6:**
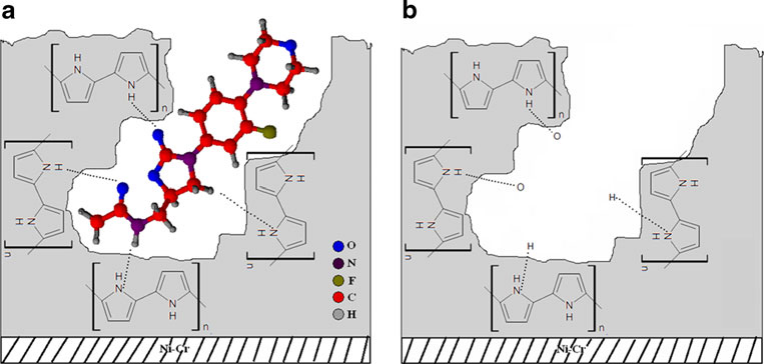
Schematic representation of **a** imprinting and **b** removal of LIN from imprinted polypyrrole modified stainless steel electrode

Based on these results, one can concluded the characteristic broadening bands at wavenumber values of about $\overline {{v}} = {3,45}0\;{\text{c}}{{\text{m}}^{{ - {1}}}}$ is associated with the formation of intermolecular hydrogen bonds between the atoms of hydrogen and sulfur present in the PTh structure and functional groups of the template, respectively. The obtained IR spectra of synthesized polymer SPME fibers confirmed the presence of functional groups, characteristic of these coatings and additionally, those characterized for the template (LIN). In the structures of the functional monomer, –N–H of PPy is hydrogen bond acceptor while –C=O of LIN is hydrogen bond donor. As shown in “Recognition mechanism of the MISPME fibers” in Fig. 6, with the addition of LIN to PPy solution during the electrochemical polymerization, the effect of hydrogen bonding occurs. The consequence of linezolid presence in the polymer matrix was to broaden the absorption band in the range between $\overline {{v}} = {3,7}00\,{\text{and}}\,{2,8}00\;{\text{c}}{{\text{m}}^{{ - {1}}}}$. It has been observed in parallel that the intensity of the multiplet in the range between $\overline {{v}} = {1,3}00\,{\text{and}}\,{9}00\;{\text{c}}{{\text{m}}^{{ - {1}}}}$, characteristic of the ion doping, also was changed. To sum up, the described characteristic vibration bands are responsible for creating the appropriate intermolecular interactions with the polymer particles (PPy, PTh, or PMeTh) of absorbent material pattern and thus the subsequent formation of fingerprints in the polymer matrix.

#### Recognition mechanism of the MISPME fibers

Molecular recognition ability is dependent on several factors, e.g., shape complementarities, functional groups, and finally, environment. For shape complementarities, the imprinted polymers should be rather rigid to preserve the stereo structures of the cavities after splitting off the template. From the general mechanism of MIP binding sites formation, applied functional monomers are responsible for the binding interactions in the imprinted binding sites. Moreover, the quantity and quality of the recognition sites in MIPs are a direct function of the mechanisms and extent of the monomer–template interactions present in the pre-polymerization mixture.

During the electrodeposition of pyrrole, linezolid template molecules are trapped into the polymer matrix as a result of these molecules ability to interact with the pyrrole units. Figure 6 shows a schematic representation of imprinting and removal of linezolid from linezolid imprinted polypyrrole SS electrode. From a theoretical point of view, at least two hydrogen bond formations are possible. The first one is between the oxygen atom in the C=O group of the linezolid molecule and the hydrogen atom in the N–H group of the pyrrole units. The second is in the hydrogen in the hydroxyl group of LIN molecule and the nitrogen atom of the N–H group of pyrrole units.

In order to remove the entrapped template, different methods were applied: electrodesorption conditions performed by cyclic voltammetry (oxidation–reduction of the template in polymer), the use of such strong solvents interacts with polymer and causing coating swelling necessary for the template release. Just to remove the template inside the SPME coating, overoxidation process was used in our work.

Such proposed sorption mechanisms of the drug (LIN) on the polymer surface material were also prepared for PTh and PMeTh MIP-coated SPME fibers. These diagrams showed the model structure of the hydrogen bonds formed between sulfur or hydrogen atoms as the polymer units and functional groups of linezolid. In addition, spatial matching was presented, which was observed between the drug molecule and the porous structure of sorption SPME coating.

Because of similar weight and significant structural relationship, another drug used for this study was amoxicillin. The use of similar chemical compounds in the adsorption and desorption processes was designed to evaluate and possibly improve the selectivity of the material with an affinity to particular molecule (MIP). Daptomycin is a macrocyclic compound, whose structure excludes the possibility of drug adsorption on the fiber with a much smaller imprint dedicated to such compounds like linezolid (“Human plasma samples”).

### Application of the SPME method in the analysis of linezolid from synthetic body fluid and human plasma samples

#### Synthetic body fluid (SBF)

**Fig. 7:**
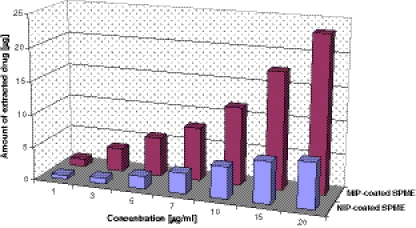
Extraction amounts of linezolid with PPy/MIP- and NIP-coated SPME fibers in the whole concentration range

Experiments performed in SBF solutions allow to get a dependence, which indicates the sorption capacity of linezolid to the applied SPME coatings. The results presented in Fig. 7 reveal the highest extraction abilities of the target compound on the PPy/MIP SPME surface. The amounts of the extracted drug at the lowest (1 μg/ml) and the highest (20 μg/ml) concentrations range from 0.96 to 5.09 and from 1.17 to 23.4 μg for PPy/NIP- and MIP-coated SPME fibers, respectively. Correlation coefficients (*R* ^2^) for target compound range from 0.9746 to 0.9966 and from 0.9655 to 0.9992 in the case of PPy/NIP- and MIP-coated SPME fibers, respectively. The relative standard deviations (RSDs) calculated for investigations in SBF solutions which were in the range 1.34–4.09% showed that adsorption repeatability for both kinds of SPME fibers was very high.

#### Human plasma samples

**Fig. 8:**
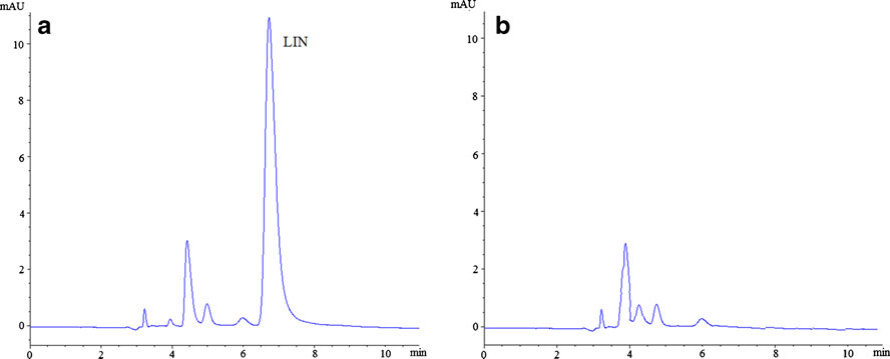
Representative MIP-coated SPME-HPLC-UV chromatograms of linezolid: **a** blank human plasma spiked with 1 μg/ml of linezolid (LIN); **b** blank human plasma

Extractions of linezolid from human plasma samples were performed using all SPME sorbent materials. The chromatograms of blank human plasma and spiked plasma after MIP-coated SPME process are shown in Fig. 8.

The results presented in the Table 3 show changes of the extracted amount of linezolid depending on the concentration of linezolid in human plasma samples. The highest extraction efficiency was obtained when using PPy/MIP SPME coatings. Some similarities in the amount of extracted linezolid between PTh/MIP and PMeTh/MIP which were observed are presented in the Table 3. The amounts of extracted drug with the use of these SPME fibers were on the lowest level compared with PPy/MIP.

**Table 3:** Extraction of linezolid from human plasma with the use of different adsorption conditions for polypyrrole (PPy), polythiophene (PTh), and poly(3-methylthiophene) MIP-coated SPME fibers (*n* = 3)

Medium for SPME extraction	Fiber	Slope	Intercept	*R* ^2^	RSD [%]
Human plasma spiked with linezolid (1–20 μg/ml)	PPy/MIP	0.4291	1.5281	0.9922	1.63
	PTh/MIP	0.3785	1.6383	0.9919	2.19
	PMeTh/MIP	0.419	0.822	0.9866	2.57
Human plasma spiked with linezolid (1–20 μg/ml) and amoxicillin (c_AMO_ = 15 μg/ml)	PPy/MIP	0.393	0.9474	0.9856	1.84
	PTh/MIP	0.349	1.0563	0.9399	2.08
	PMeTh/MIP	0.368	1.993	0.9671	2.98
Human plasma spiked with linezolid (1–20 μg/ml) and daptomycin (c_DAPTO_ = 15 μg/ml)	PPy/MIP	0.4125	0.878	0.9866	2.16
	PTh/MIP	0.3682	1.9383	0.9345	3.01
	PMeTh/MIP	0.3873	1.7282	0.9692	3.79

Linear regression parameters calculated for PPy, PTh, and PMeTh MIP-coated SPME fibers in dependency with a different composition of medium for extraction of LIN are presented in Table 3. In case of polypyrrole fibers, the highest correlation coefficients (*R* ^2^) were calculated in range between 0.9856 and 0.9922. The best extraction efficiencies were the highest for PPy/MIP SPME coating and were allowed to obtain good correlations coefficients (*R* ^2^) in comparison with the other applied MIP-coated SPME fibers. RSDs calculated for all applied SPME coatings in all medium for adsorption are similar also in range from 1.63% to 3.79%. Table 4 compares extraction efficiencies in synthetic body fluid to the human plasma samples. Polypyrrole MIP-coated SPME fibers provide better sorption efficiencies than PTh/MIP or PMeTh/MIP SPME fibers for both studied matrices.

**Table 4:** Comparison of target compound amount extracted by PPy/MIP vs. PTh/MIP vs. PMeTh/MIP fibers from SBF and human plasma samples (*n* = 3)

Concentration [μg/ml]	Amount of extracted linezolid [μg] PPy/MIP vs. PTh/MIP vs. PMeTh/MIP	
	Synthetic body fluid	Human plasma
1	1.17/0.89/0.96	0.95/0.81/0.84
3	3.51/2.66/2.88	2.84/2.43/2.52
5	5.85/4.43/4.8	4.73/4.05/4.2
7	8.02/6.20/6.74	6.54/5.67/5.89
10	11.7/8.85/9.6	9.45/8.1/8.4
15	17.55/13.28/14.4	14.18/12.15/11.25
20	23.4/17.7/19.2	18.9/16.2/16.8

### Analytical validation of the developed method

The specificity was evaluated by comparison to blank and spiked human plasma samples (Fig. 8). Linezolid was eluted at 6.92 min. No apparent interference was observed in the matrix. Calibration curve was established by analyzing a series of spiked samples as described in “Preparation of stock and standard solutions”. The calibration curve $\left( {y = ax + b} \right)$ was constructed by plotting peak area ratio (*y*) against analyte concentrations (*x*). The calibration curve exhibited good linearity with correlation coefficients (*R* ^2^) of 0.9996 in broad dynamic range. The limit of detection was equal 0.029 μg/ml. The limit of quantification was 0.086 μg/ml. Precisions were determined by analyzing three different concentrations of LIN samples (five replicates for each concentration) for three consecutive days. Within- and between-batch precisions were evaluated by RSD, which ranged from 3.4–7.5% to 3.8–11.2, respectively (Table 5). Accuracy was calculated as a relative error (RE) by following formula: ${\text{RE}}\left( \% \right) = \left[ {\left( {{{\text{c}}_{{{ \det }}}} - {{\text{c}}_{\text{nom}}}} \right)/\left( {{{\text{c}}_{\text{nom}}}} \right)} \right] \times {1}00$, where c_nom_ represented the nominal concentration and c_det_ represented the mean value of detected concentrations. The calculated accuracy values for three concentrations were in ranges between 1.4–4% and 4–11.4% for SBF and human plasma, respectively. The above data met the FDA requirements for bioanalytical method validation, indicating this method was acceptable for pharmacokinetics studies as well [33].

**Table 5:** Precision and accuracy of linezolid (LIN)

Medium	Concentration added (μg/ml)	Within-batch (*n* = 5)			Between-batch (*n* = 3)		
		Concentration measured (μg/ml, mean ± SD)	Precision (%, RSD)	Accuracy (%, RE)	Concentration measured (μg/ml, mean ± SD)	Precision (%, RSD)	Accuracy (%, RE)
Synthetic body fluid	1	0.96 ± 0.67	5.5	−4.0	0.93 ± 1.23	11.2	−7.0
	7	7.1 ± 0.49	7.5	1.4	6.7 ± 1.10	10.6	−4.3
	15	15.3 ± 0.36	3.4	2.0	14.9 ± 0.70	7.3	−0.7
Human plasma	1	0.94 ± 0.72	4.8	−6.0	0.89 ± 1.02	6.6	−11.0
	7	6.2 ± 0.53	4.8	−11.4	6.1 ± 0.89	3.8	−12.9
	15	14.4 ± 0.39	4.6	−4.0	14.1 ± 0.48	5.4	−6.0

Calibration curves were set up for target SPME coatings. A good linearity was found for each of them in the whole concentration ranges. The precision was also satisfactory, with RSD values lower than 12% for each of the tested SPME coating. Calibration curve parameters, presented in Table 3, showed good correlation coefficients (*R* ^2^) from 0.9345 to 0.9922. Additionally, differences in slopes presented in Table 3 proved the higher selectivity of PPy/MIP SPME coating in comparison with the other applied coatings. The parameters that described the concentration of extracted linezolid vs. concentration in output plasma samples were collected and are presented in Table 5. To complete the validation method, accuracy and RSD values for three concentrations of linezolid and applied SPME coatings were also calculated and are shown in Table 5. The values of method accuracy are compatible with the relevant FDA guidelines which suggested the mean value to be within 15% of the actual value [33].

Stability of LIN in SBF and human plasma samples were tested at three concentration levels by a series of experiments. The results of stability tests for biological samples: autosampler, freeze and thaw, short-term, and preliminary long-term at two storage temperatures are presented in Table 6.

**Table 6:** The stability of linezolid in human plasma under different conditions

	Amount found (mean ± SD %)		
	1 μg/ml	7 μg/ml	15 μg/ml
Short-term stability	1.2 ± 0.82	6.9 ± 0.39	15.1 ± 1.21
Long-term stability	1.3 ± 1.1	7.2 ± 1.29	15.2 ± 4.21
Freeze–thaw stability	1.2 ± 0.48	7.3 ± 2.11	14.9 ± 1.3
Post-preparative stability	1.1 ± 0.36	7.1 ± 0.94	15.3 ± 0.87

For each concentration level, there were acceptance criteria falling within the range of 85–115%. In addition, evaluated were the respective stability tests that confirmed the stability of linezolid in the stock and working solutions at 1–4 °C after 1 month. The target compound was proven to be stable both in stock solution (at −4 °C) and in SBF, as well as in human plasma samples (at −20 °C) after long-term storage (for 1 month). These data indicated the analyte was stable during sample preparation and chromatographic measurements.

The proposed analytical procedure seems to be characterized by specific selectivity to linezolid and could be used for the trace analysis of other oxazolidinones in bio-fluid samples in routine analysis, what is very important in clinical studies.

## Conclusions

The presented study demonstrates that the MIPs application as sorbent for MIP-coated SPME procedure are shown sufficient enough, rapid, and reproducible in simultaneous preconcentration and clean-up of biological fluids especially human plasma samples. In this paper, the electrochemical polymerization method was improved to prepare novel linezolid MIP-coated SPME fibers. The MIP coatings were homogeneous and porous. Both fiber preparation and extraction were reproducible. The extraction efficiencies of linezolid with the MIP-coated fibers were markedly higher than with the NIP-coated fibers. A MIP-coated SPME-HPLC method for the simultaneous monitoring of linezolid was established. The detection limit for linezolid was 0.029 μg/ml and could meet therapeutic monitoring of trace linezolid in the serum and plasma. The linezolid MIP-coated fibers are suitable for the extraction of trace oxazolidinones in the complicated biologic samples. Additionally, the MIP-coated SPME methodology followed by HPLC with UV and MS is easy, reliable, and sensitive at the trace level, requiring a low sample volume, and seems to be a good analytical alternative to routine quality control for biomedical analysis. The method is simple and sensitive, and can be used as an alternative tool to effectively separate and enrich the target compounds by SPME-liquid chromatography (LC)/MS.
